# Preclinical research in obesity-associated metabolic diseases using in vitro, multicellular, and non-mammalian models

**DOI:** 10.1007/s13105-025-01130-6

**Published:** 2025-10-24

**Authors:** Paula Aranaz, Marina Clavel-Millan, Katherine Gil-Cardoso, Maitane González-Arceo, David Hernández-González, Francisco Les, Jérôme Salles, Ez-Zoubir Amri, José M. Arbones-Mainar, Claude Atgié, Frédéric Capel, Arnaud Courtois, Xavier Escoté, María José García-Barrado, Stéphanie Krisa, Víctor López, Fermín I. Milagro, María P. Portillo, Silvia Lorente-Cebrián

**Affiliations:** 1 Department of Nutrition, Food Sciences and Physiology, Center for Nutrition Research, University of Navarra, Pamplona, 31008 Spain; 2 Navarra Institute for Health Research (IdiSNA), Pamplona, 31008 Spain; 3 Adipocyte and Fat Biology Laboratory (AdipoFat), Hospital Universitario Miguel Servet, Zaragoza, 50009 Spain; 4 Aragón Health Research Institute (IIS-Aragon), Zaragoza, Spain; 5 Eurecat, Centre Tecnològic de Catalunya, Nutrition and Health Unit, Reus, 43204 Spain; 6 Nutrition and Obesity Group, Department of Nutrition and Food Science, University of the Basque Country (UPV/EHU) and Lucio Lascaray Research Institute, Vitoria, 01006 Spain; 7 Neuroendocrinology and Obesity Team, Faculty of Medicine, INCyL and IBSAL, University of Salamanca, Salamanca, Spain; 8 Department of Pharmacy, Faculty of Health Sciences, Universidad San Jorge, Zaragoza, 50830 Spain; 9 Instituto Agroalimentario de Aragón-IA2, CITA-Universidad de Zaragoza, Zaragoza, 50013 Spain; 10 Unité de Nutrition Humaine (UNH), UMR1019, Université Clermont Auvergne, INRAE, CRNH Auvergne, Clermont-Ferrand, France; 11 CNRS, Inserm, Adipocible Research Study Group, Institute Biology Valrose (iBV), Université Côte d’Azur, Nice, France; 12 CIBER Fisiopatología Obesidad y Nutrición (CIBERObn), Instituto Salud Carlos III, Madrid, 28029 Spain; 13 Instituto Aragonés de Ciencias de la Salud, Zaragoza, 50009 Spain; 14 Université de Bordeaux, Bordeaux INP, CNRS, Institut CBMN, UMR 5248, Equipe Val’Actif (Valorisation d’Actifs), Pessac, 33600 France; 15 UMR OEnologie (UMR 1366, INRAE, Bordeaux INP), Institut des Sciences de la Vigne et du Vin, Université de Bordeaux, Villenave d’Ornon, 33882 France; 16 Nutrition and Metabolic Health Research Group, Department of Biochemistry and Biotechnology, Rovira i Virgili University (URV), Reus, 43201 Spain; 17 Institute of Health Pere Virgili (IISPV), Reus, 43204 Spain; 18 Center of Environmental, Food and Toxicological Technology—TecnATox, Rovira i Virgili University, Reus, 43201 Spain; 19 Department of Pharmacology, Physiology and Legal and Forensic Medicine, Faculty of Health and Sport Science, University of Zaragoza, Zaragoza, Spain

**Keywords:** Adipocytes, Enterocytes, Skeletal muscle cells, Tissue explants, Spheroids, Organoids, Zebrafish, *Drosophila*, *Caenorhabditis elegans*, Gastrointestinal simulator

## Abstract

Addressing the physiological effects of bioactive compounds in metabolic diseases (i.e., obesity, diabetes, liver steatosis) and establishing their mechanisms of action have been a major interest for the last decades. However, methodologies that can be applied to achieve this can vary greatly, leading to a limited type of information. Thus, the accuracy, robustness, reliability and potential (human) translation are highly reliant on the experimental design and selected methodological models. This review presents an update exploring the main features, advantages and disadvantages of most important pre-clinical models used at the present time to study the effects of bioactive compounds on metabolic diseases. Moreover, future challenges in developing new methods are also depicted. In vitro models (enzyme assays and standard two-dimensional cultures of adipocytes, skeletal muscle cells) are intrinsically well established and constitute the first choice and most widely used methods to study bioactive compounds in metabolic diseases. However, novel models such as three-dimensional cultures (spheroids, organoids) are also starting to emerge and complement traditional culture systems. Models of small organisms (*C. elegans*,* D. melanogaster*) and non-mammal vertebrates (*D. rerio*) represent a scientific advantage and a middle-step before traditional mammalian models (rats and mice). This article provides extensive information and a critical overview of a wide range of methods that represent present and future avenues towards a further understanding of metabolic diseases. Combining and developing new methods will be key for future progression on the effects of bioactive compounds on metabolic diseases, as well as to minimize the use of mammalian models due to ethical reasons.

## Introduction

Animal models are used in experimental research to advance in physiology and physiopathology of many diseases and/or to explore new treatments. Their use is supported by several reasons, including the possibility of experimenting under controlled situations, the possibility of mimicking biological conditions of human diseases, which allows translating the knowledge gained in an animal model to human patients, and the ethical distinction made between humans and animals [6, 98].

However, a growing awareness of the concept of animal rights has involved the necessity to reduce their use in those research areas where alternative in silico or in vitro methods are available. For example, recent advances in stem cell biology and microfluidics are enabling big advances in patient- and disease-specific drug testing; based on these technologies, it is possible that in a close future, organ-on-a-chip and multi-organ-on-a-chip will lead to the replacement of animal testing [202]. But in the meantime, it is necessary to find models that, with low cost and easy handling and maintenance, can help to perform preclinical research on human diseases. For this purpose, it is crucial to identify and select the most appropriate model to replicate the condition that is under investigation and to address specific research questions [43]. The present review is focused on the main models that can be used in the study of bioactive compounds in metabolic diseases, including the effects on intestinal, skeletal muscle and adipose tissue metabolism, as well as the pathophysiological mechanisms per se that lie behind disease. The models that will be analysed in depth are in vitro enzyme inhibition bioassays, cellular models of enterocytes, skeletal muscle cells, and adipocytes (including stem-cells, co-cultures, and tissue explants), spheroids, organoids, and whole animal (small organisms) models like *Caenorhabditis elegans*, *Drosophila melanogaster*, and *Danio rerio*.

## In vitro enzyme inhibition bioassays

Understanding the mechanisms of action of bioactive compounds is crucial in drug discovery and development. Enzyme activity models serve as invaluable tools in elucidating these mechanisms, particularly for new or poorly understood bioactive compounds. By simulating enzyme reactions in controlled environments, these models provide insights into how bioactive molecules interact with specific targets, offering advantages over traditional animal or human studies. Metabolic syndrome is a cluster of disorders occurring together, increasing the risk of heart disease, diabetes, obesity, and other health conditions [139]. Hence, numerous enzymes participate in the pathophysiological processes behind metabolic syndrome features. Some important enzymes whose inhibition could be related to the prevention of the metabolic syndrome and associated complications include pancreatic lipase, glucosidase, amylase, dipeptidyl peptidase 4 (DPP4), and others [63]. These enzymes are involved in carbohydrate metabolism, fat metabolism, incretin control, or other physiological markers. Prevention of metabolic syndrome is achieved through changes in heart-healthy lifestyle, such as a healthy diet, weight loss, stress management, regular physical activity, and smoking cessation [99]. However, the knowledge of bioactive compounds affecting metabolic pathways related to the pathophysiology of these diseases can aid the aforementioned strategies as many of the bioactive compounds can be acquired through dietary habits or the consumption of certain nutraceuticals and functional foods.

### Lipases

Pancreatic lipase is an enzyme produced by the pancreas that helps to break down fat in the small intestine. Therefore, inhibiting pancreatic lipase is a strategy to combat obesity. When pancreatic lipase is inactive, triglycerides are not hydrolyzed, and the intestinal epithelium cannot absorb fat for subsequent storage in the body. To measure the inhibitory capacity of a compound against lipase, a colorimetric method using porcine pancreatic lipase, p-nitrophenyl butyrate (pNPB) as a substrate, and buffer to maintain physiological conditions can be performed [188]. To inhibit pancreatic lipase type II from porcine pancreas, a 96-well microplate reader can be employed. Each well should contain 40 µL of the test sample and 40 µL of type II lipase from porcine pancreas (2.5 mg/mL in Tris buffer, pH 7.0). After a pre-incubation period of 15 min, 20 µL of a 10 mM pNPB solution is added as the substrate. The mixture is then incubated for an additional 15 min at 37 °C. Enzymatic activity is determined by measuring the absorbance at 405 nm. Orlistat could be used as a positive control for lipase inhibition. This reference inhibitor is a Food and Drug Administration (FDA) and European Medicines Agency (EMA)-approved reference medication against obesity that inhibits pancreatic lipase. However, inhibiting the lipase enzyme can lead to a series of gastrointestinal non-desired effects. These can range from severely affecting fat digestion, malabsorption, bloating, to fatty diarrhea. Bioactive compounds, especially polyphenols, can be an alternative to orlistat [26]. Flavonoids, bioactive compounds of great relevance due to their interactions with digestive enzymes, have been shown to be effective in inhibiting pancreatic lipase. Some of these are quercetin, rutin, luteolin, catechin, or hesperetin. Compounds such as ellagic acid, punicalagins, or urolithins from pomegranate are also good inhibitors of this enzyme [110].

### Peptidases

DPP4 is a membrane-bound integral enzyme with protease activity that plays a significant role in the degradation of incretins, bioactive molecules like glucose-dependent insulinotropic polypeptide (GIP) and glucagon-like peptide-1 (GLP-1). It plays a significant role in the degradation of bioactive molecules and is involved in the pathogenesis of ventricular dysfunction, diabetes, and inflammation [116]. Diabetic rodents expressing DPP4 exhibit increased local activity of this protein at the cardiac level, which is associated with increased diastolic stiffness and relaxation time constant. Additionally, diabetic rodents expressing DPP4 are characterized by reduced phosphorylation of the endothelial nitric oxide synthase (eNOS) signaling pathway, indicating endothelial dysfunction. DPP4 has also been linked to diabetes and inflammation. DPP4 inhibitors, such as saxagliptin, alogliptin, sitagliptin, vildagliptin, and linagliptin, are used in the treatment of type-2 diabetes mellitus (T2DM). These drugs act on the DPP4 enzyme, nullifying its inhibitory action on incretin hormones, thus increasing the half-life of these hormones and improving blood glucose control. To evaluate the inhibitory activity of bioactive compounds against this enzyme, a fluorometric method in a 96-well plate could be performed, and H-Gly-Pro conjugated to aminomethylcoumarin can be used as a substrate [112]. Each well contains a mixture of diluted assay buffer, DPP4 enzyme, and either solvent or inhibitor, followed by the addition of a fluorogenic substrate. Plates were incubated at 37 °C for 30 min and fluorescence was measured at an excitation wavelength of 350–360 nm and an emission wavelength of 450–465 nm. Sitagliptin is typically used as the reference inhibitor. Tests with this enzyme and bioactive compounds are relatively new. However, some polyphenols have shown inhibitory capacity in this enzyme. Again, ellagitannins from pomegranate are good inhibitors of this enzyme [110]. Also, chlorogenic acid, or anthocyanins such as cyanidin from blueberry, cranberry or sour cherry, are capable of inhibiting DPP4 in a dose-dependent manner [36–39].

Recently, novel anti-obesity drugs targeting gut-derived hormones have emerged as promising therapeutic agents. Among them, dual agonists of glucose-dependent insulinotropic polypeptide (GIP) and glucagon-like peptide-1 (GLP-1) receptors, such as tirzepatide, or single GLP-1 analogs (semaglutide) have shown significant efficacy in promoting weight loss and improving glycemic control [9, 92]. These incretin-based therapies enhance insulin secretion, reduce appetite, and delay gastric emptying [88, 137]. Although their mechanism is not based on direct peptidase inhibition, their relevance in obesity treatment highlights the importance of targeting peptide-mediated metabolic pathways, complementing the role of peptidase inhibitors in modulating metabolic processes.

### Glycosidases

α-amylase and α-glucosidase are enzymes related to digestion helping to hydrolyse carbohydrates. α-amylase is secreted by the salivary glands and the pancreas and is responsible for hydrolysing glycogen and starch into small oligosaccharides. Subsequently, intestinal α-glucosidases, which are mainly located in the brush border of enterocytes, promote the release of glucose units from smaller units of maltose and other oligosaccharides [106]. These enzymes have been a therapeutic target in the treatment of T2DM since acarbose is the oral antidiabetic drug whose mechanism of action is based on the inhibition of these enzymes. Although the FDA approved the first inhibitor of these enzymes in 1995, the fact that it produces digestive complications such as flatulence and abdominal distention means that its use in clinical practice is very restricted. A large number of studies suggest that some of the natural compounds present in plants and foods act as α-amylase and α-glucosidase inhibitors. Specifically, flavonoids are one of the phytochemical groups mostly involved in this mechanism of action as natural substances with antidiabetic properties. As a consequence of this enzymatic inhibition, a delay in the digestion of carbohydrates occurs, which leads to a decrease in the amount of glucose that reaches the bloodstream. In addition, many flavonoids exert actions on different molecular pathways related to glycaemia but also exert antioxidant, anti-glycaemic, and anti-inflammatory actions, making them bioactive compounds of interest in the management and prevention of metabolic complications related to hyperglycaemia. One of the examples could be the flavonoid baicalin, a flavonoid present in the species *Scutellaria baicalensis*, used in traditional medicine for various diseases related to inflammatory processes, hypertension or diabetes [191]. A large body of evidence also suggests that quercetin is a flavonoid with antidiabetic activity acting as an α-glucosidase inhibitor, being widely distributed in foods of plant origin [28, 177]. Among the flavonoid group, anthocyanins, a class of flavonoids, are present in significant quantities in numerous red or blue berry-like fruits such as blueberries, strawberries, raspberries, currants and grapes; in these fruits, anthocyanins act as plant pigments but from a nutritional point of view they act as bioactive compounds and have also demonstrated inhibitory mechanisms of amylases and glucosidases as responsible for the protective activity they present in hyperglycaemia and T2DM [111]. Inhibition of α-glucosidase commercially available from *Saccharomyces cerevisiae* can be evaluated at 405 nm in 96-well microplates; the samples must be incubated with the enzyme (1 U/ml) for 10 min at room temperature and the substrate (4-nitrophenyl α-d-glucopyranoside or NPG) is added and incubated at 37 °C before reading the absorbance. In the case of α-amylase, the inhibition can be quantified using enzyme available from porcine pancreas in 96-well microplates at 540 nm; in this case the tested compounds or samples are pre-incubated with the enzyme (2 mg/ml) in microtubes at 37 °C for 5 min and then with a 1% starch solution for another 10 min; 200 µL of 1% 3,5-dinitrosalicylic acid as colour reagent and 50 µL of 1 M NaOH is added for 5 min heated at 100 °C; once the reaction was finished, the microtubes must be cooled in a bath of cold water and absorbance measured at 540 nm after transferring 200 µL of the solution from each microtube into the microplate.

### Protein-tyrosine phosphatases

Protein tyrosine phosphatase 1B (PTP1B) is also involved in the regulation of glycaemia as this enzyme acts as a negative regulator of insulin and leptin receptors [120, 228]. PTP1B has been proposed as an important pharmacological target for the treatment of type 2 diabetes because it induces the dephosphorylation of the insulin receptor. When this occurs, the glucose transporter type 4 (GLUT4) is not transported into the cell surface and the glucose cannot be uptaken into the cell. Nevertheless, natural PTP1B inhibitors act as antidiabetic agents. Although there are not current marketed drugs acting as inhibitors of this enzyme, many natural products from different chemical structures (polyphenols, terpenoids and alkaloids) have shown bioactivity in this target. *Gymnena sylvestre* has been used as a traditional antidiabetic medicinal plant and some of its oleanane triterpenoid have shown inhibitory activity of the PTP1B enzyme. Certain polyphenols have also shown activity against this enzyme; for example, flavonoids from *Psoralea corylifolia*, eriodyctiol and phenolic acids from *Morus alba* exerted significant activity [120]. Natural PTP1B inhibitors seem promising although their clinical applications remain unclear. In this case, the enzyme (human recombinant PTP1B), the substrate (p-nitrophenyl phosphate or NPP), a redox reagent for stabilizing thiol groups (dithiothreitol or DTT) and the samples must be dissolved in 50 mM Tris and 50 mM Bis-Tris buffer containing 100 mM NaCL and adjusted to pH 7; the potential inhibitors must be incubated with a 1.5 mM NPP and 6 mM DTT solution for 10 min at 25 °C and then 50 µL of 0.001 µg/µL PTP1B stock solution in phosphate buffer is added being absorbance monitored at 405 nm every 30 s for 10 min.

These enzyme activity models offer several advantages over traditional experimental approaches, allowing precise control of experimental conditions, including substrate concentration, pH, and temperature, ensuring reproducibility and reliability of results. These enzyme activity assays can be automated, allowing rapid screening of large compound libraries to detect potential bioactivity. Furthermore, compared to studies in animals or humans, enzyme activity models are more cost-effective and ethically accepted, requiring fewer resources and avoiding ethical concerns related to animal experimentation. These models provide detailed mechanistic information on how bioactive compounds interact with specific enzymes, facilitating the design of more effective therapies. The in vitro enzyme inhibition assays may have some limitations regarding the pharmacokinetics of the tested substances which involves aspects of bioaccesibility, bioavailability or bioconversion into other metabolites. However, it should be stated that the bioactive compounds that are tested in these in vitro assays will not be the real metabolites that could eventually reach functional enzymes in an in vivo context providing that polyphenols are eventually metabolized before reaching target tissues.

## Intestinal cell cultures

### The benefits of cellular models

Cellular models are used to study, in a simplified context, complex in vivophenomena. As they are simpler to a whole organ or a whole organism, they represent useful and interesting tools for studying the physiological or pathophysiological processes underlying disease development, progression and/or treatment. Some in vitro cellular models represent valuable alternative methods to animal experiments, with respect to the 3R rules (Replacement, Refinement and Reduction), that are recognized in basic but also in applied pharmaceutical and cosmetic research [152].

### Limitations of cellular models

Nevertheless, limits to cellular models are integrated in their definition. They do not reflect interactions of all cell populations in an organ, and especially it is difficult to consider the interactions between several organs, to integrate the hormonal or nervous regulations or to consider the motility of specific organs of the gastrointestinal tract. Sometimes the primary culture of organ-derived cells or the use of immortalized cells lose their differentiated functions that represent a major difficulty to the study of certain specific functions of the organ that the model is devoted to. Nevertheless, there is a need to develop intestinal models as part of the screening tools of bioactive compounds for subsequent use in in vivo studies before their administration to humans.

### Development and examples of intestine cellular models to study the beneficial effects of bioactive compounds related to intestinal functions

The intestine integrates several functions, the main are digestion and absorption of nutrients, but also, their metabolism and degradation, during the absorption across the enterocytes. These functions of digestion and absorption of nutrients are associated to secretory and immune functions. Thus, the intestine is constituted of heterogeneous population of cells that interact each together and with bacteria in dynamic conditions [198]. Other features that should be integrated are the architecture of the intestine organized in finger-like microvilli and the shear stress associated with the motility of the intestine. Therefore, the use of the ideal cellular models to represent the intestine should contain the representatives cell types of this organ (epithelial, secretory and immune cells) in a fluidic system that provide some shear stress and of great importance, the intestine microbiota. This last point could be very important, as it is known that many potential bioactive compounds (polyphenols, for example) can undergo transformations by intestinal bacteria, producing specific metabolites which could also be absorbed to act on a target organs [71].

Among several cellular models that are available for the study of the permeability and metabolism of bioactive compounds, the Caco-2 cell line probably represents the most widely used cell type to model intestinal barrier. They are derived from a human colorectal carcinoma [181]. In vitro, this cell line forms a monolayer of cells. Upon reaching confluence, Caco-2 cells have the intrinsic ability to initiate spontaneous differentiation. At day 21 post-confluence, the cell surface develops typical enterocytes-like morphology (regular microvilli and full structural polarization). The monolayer develops characteristics very similar to the intestinal barrier. In the beginning of the culture cell process, before differentiation, this cell culture model was often used in food and pharmaceutical research for a quick screening of the acceptability of potential new molecules of interest, leading to the establishment of a maximal tolerable concentration. Moreover, after the differentiation process (day 21 post-confluence), using a Transwell^®^ system, the study of the transport and the metabolism of bioactive compounds across the intestinal barrier (absorption) can be performed. Nevertheless, this mono-cell type culture does not include some features as the presence of mucus layer, the interactions with immune, endocrine, or stromal cells, or the motility of the intestine. In addition, this model lacks on the interaction with gut microbiota.

In order to circumvent these issues, several groups have developed more sophisticated models that integrate two or three types of cell lines co-cultured. In such models, the Caco-2 cells are co-cultured either with HT-29/MTX cells (mucus producing cells) [113], optimised by fatty acid pre-treatment to restore the original histological multilayer structure of the intestinal barrier [20], or with intestinal M-cells (Microfold cells that are found in the gut-associated lymphoid tissue) [8]. Other groups developed cells scaffolds to create a 3D architecture that enable cells to proliferate in 3D villi-like structures, and obtained a cellular model with barrier function similar to in vivo situation, and interestingly with enhanced metabolic activity [221] compared to conventional 2D cellular cultures. This metabolically functional barrier is of great interest to study the absorption of polyphenol into blood circulation either in their native form or their metabolites produced by intestine.

In the literature, several examples described the usefulness of those in vitro models to study the effects of bioactive compounds. Since bioactive compounds, such as polyphenols, could exert their beneficial effects through their own or through their metabolites, the study of their metabolism is of great relevance. This optimised co-culture model could represent an efficient solution since many cell types, especially primary cells or immortalized cells, loose their metabolic function due to dedifferentiation processes. For all compounds orally ingested, the so-called first pass metabolism, as part of the global absorption-distribution-metabolism-excretion (ADME) process, involves the gut and the liver that could greatly affects the bioavailability and biological effects of a bioactive compound. Some authors succeeded in the development of a microfluidic gut-liver chip that can reproduce this first pass metabolism [23, 50]. This metabolic activity of the two derived cell lines from both organs was maintained. The use of this device to study the metabolic profile of apigenin showed that majority of metabolic transformation of apigenin was exerted by the intestinal cell similar to what was observed inin vivo situation [195].

### Integration of bacteria into cellular models

Another feature of the intestine is the presence and the interaction of bacteria with enterocytes or immune cells. The importance of gut microbiota to metabolize some nutrients or bioactive compounds, to metabolize and eliminate exogenous contaminants, to educate our immune system and to maintain gut barrier integrity is indisputable. These aspects are well documented by recent studies performed to understand the onset event, for example, of the chronic inflammatory intestinal pathologies [149]. To integrate bacteria into cellular models, one strategy is to co-culture intestinal cell lines with bacteria at the luminal surface of cells monolayer. This strategy is hampered by the fact that most bacteria are anaerobic, whereas cells need oxygen to develop. It is necessary to succeed in making these two entities co-exist within the same culture. Such system was developed by Shah et al., the so-called Humix^®^ system that used a modular microfluidics device and allow the co-culture of human intestinal cells and microbial cells [171].

As for the permeability or the metabolism studies, several examples in the literature described the usefulness of such models to study the importance of gut microbiota. As an example, the beneficial effects of polyphenols have recently been illustrated by desaminotyrosine, a gut metabolite derived from the degradation of quercetin, luteolin or apigenin by *Clostridium orbiscindens*. This metabolite enhances type I IFN signalling and rescues influenza-infected mice. Authors observed that, after oral administration of polyphenols, mice produce nanomoles of desaminotyrosine *per* gram of faeces and picomoles of desaminotyrosine in their blood, thus highlighting that specific metabolite derived from gut microbiota can exert distal effects [189].

Another example is provided by the urolithins that are ellagitannins and ellagic acid-derived gut microbiota metabolites. Several studies have highlighted their role in the preservation of a functional gut barrier. Indeed, high fat diet (HFD) is responsible of gut microbiota imbalance and damage to colon tissue that increases intestinal permeability and promotes a low grade deleterious chronic inflammation. This low-grade chronic inflammation is involved in many metabolic diseases, obesity for example. In such cases, some polyphenols could normalize microbiota imbalance and promote the development of beneficial bacteria. In addition, the polyphenols metabolites urolithins can inhibit the gut barrier dysfunction by restoring the tight junction proteins expression (claudin-1 and zonula ocludens-1). Their effects probably occurred through the AhR or Nrf2-dependent pathways [183, 205, 229].

Those observations, and many others, illustrated the beneficial role of bioactive compounds on gut functions. Those compounds exert their activities either through a direct interaction with intestinal cells or by an indirect interaction through their effects on gut microbiota or their microbial metabolites. In this context, cellular models remain essential to help us to understand their mechanisms of action. Nevertheless, as most bacterial strains are not cultivable and cell culture conditions are often not suitable with bacterial culture conditions, this represents a major limitation to this model.

To conclude, functional in vitro models of the gut currently exist and prove highly valuable in assessing the benefit-risk ratio of functional foods. They offer a viable alternative to in vivo experiments. However, much remains to be accomplished in developing comprehensive models capable of accurately mimic the complex interactions between the gut and microbiota that underlie the effects of numerous bioactive compounds.

## In vitro models of skeletal muscle

### Skeletal muscle cells

Skeletal muscle is the most abundant tissue in the human body, accounting for 40 to 50% of total body mass and plays an important function in locomotion, posture and breathing. The skeletal muscle is also a crucial contributor to whole-body metabolic homeostasis through the utilization of glucose, lipids and as a reserve of amino acids. One of its major roles is the contribution to postprandial glucose disposal, accounting for 80–85% of the glucose uptake in insulin-sensitive tissues [55].

Skeletal muscle is composed of muscle fibers grouped into bundles covered with connective tissue. Each muscle fiber corresponds to a single elongated, multinucleated muscle cell. Connective tissue promotes the passage of blood vessels and nerves which are abundant in skeletal muscle. A pool of muscle stem cells is also present, called satellite cells, able of self-renewal and differentiation to enable muscle development, maintenance and regeneration [65, 222]. Satellite cells can be isolated from muscle fragments, cultured, activated to myoblasts able to proliferate and then induced to differentiate into elongated multinucleated structures resulting from the fusion of several myoblasts, called myotubes. Current experimental policy promotes the principles of the 3Rs. Satellite cell-derived myoblast culture is a useful tool for replacement of animal experimentation in skeletal muscle studies. They are extensively used to study insulin resistance, anabolic resistance, lipotoxicity and inflammation. In this review, we present two-dimensional (2D) in vitro skeletal muscle models and their use in metabolic studies. We also introduce a more representative model of human skeletal muscles, i.e. the three-dimensional (3D) skeletal muscle cell culture model, that could accelerate the reduction of live animal experiments.

### 2D culture models

2D cell models are widely used to mimic the mechanisms and responses to natural or pharmacological therapeutics of the metabolic abnormalities associated to obesity. Regarding the contribution of skeletal muscle to metabolic homeostasis, the tissue has been extensively studied in the field of obesity and insulin resistance. Hence, intramyocelular accumulation of some lipid mediators, such as acyl-CoA, diacylglycerols and ceramides, was found to negatively regulate the response to insulin through the inhibition of PKB/Akt activation, leading to the impairment of glucose uptake and alterations in the insulin-dependent stimulation of protein synthesis, a crucial step for the maintenance of skeletal muscle homeostasis [129, 147]. Lipotoxicity corresponds to lipid-induced metabolic abnormalities and could be mimicked by the treatment of muscle cells in vitro with ceramides or long chain saturated fatty acids, notably palmitic acid. The major cell lines that are used for many years to study metabolic disorders are rat L6 and mouse C2C12 myoblasts. Differentiated adherent myotubes could be rapidly obtained from these commercial cell lines allowing experiments over a period of 3 to 7 days. Alternatively, human skeletal muscle primary cells are also widely used. Using these models, several studies were set up to explore the mechanisms linking lipid infiltration in skeletal muscle and the perturbation of its metabolic homeostasis. It has been shown that PKB/Akt activation was impaired in L6 and C2C12 myotubes exposed to ceramides or palmitic acid as in human myotubes [126]. Interestingly the detailed mechanisms differed between the two rodent cell lines [2], but the effect on insulin sensitivity closely represented the observation made with human myotubes [126]. Furthermore, it has been observed that cultured muscle cells could represent donor’s phenotypes in term of insulin resistance [1–4, 12]. Cell line’s specific transcriptomic signatures were recently identified [2] suggesting that the choice of the cell line may vary depending on study’s objective. L6 myotubes exhibited the greatest expression level of genes related to glucose uptake and oxidative metabolism. Human cells exhibited a higher expression of glycogen synthesis genes although C2C12 cells highly expressed contractile proteins [2].

Compelling results have shown that cultured myotubes could be used to evaluate the effect of drugs or nutrients against ceramide accumulation and insulin resistance, or to identify intracellular regulators [31, 32, 45, 46, 193]. Altogether, the data obtained using myotubes in vitro, coming from commercial or human samples strongly suggest that these approaches could represent a relevant methodology to study obesity-related metabolic disorders in skeletal muscle. More recently, the use of immortalized human skeletal muscle primary cells offers new possibilities to select samples according to donor’s characteristics such as age or body weight [44, 127] and perform experiments over the years.

The 2D culture models of skeletal muscle cells have several advantages such as the simplicity and low-cost of maintaining cells in culture, the short time required to obtain the fusion of myoblasts into multinucleated myotubes (2 to 3 days) and the easy use of genetic manipulation [57, 97]. Furthermore, all targeted cells are similarly exposed to tested therapeutic molecules or nutrients. Despite its wide use to study the mechanisms of development, proliferation, differentiation and metabolic disorders, these conventional 2D muscle cell culture models also have some limitations. The first is the absence of the natural microenvironment (i.e. cell-cell and cell-extracellular matrix interactions). Secondly, the detachment of the myotubes from the surface of the culture support after 7 days of differentiation due to spontaneous contractions of the myotubes prevents long-term interventional studies. Finally, the difference in architecture compared to a native muscle (unidirectional alignment of multinucleated cells) restrains the provision of relevant physiological data [75, 93, 172].

To overcome the limitations of 2D cell cultures, several 3D skeletal muscle models have been developed to recreate the structural organization and functions of adult skeletal muscle. These tissue-engineered skeletal muscles can be categorized into scaffold-free/self-assembled tissues (myospheres) or scaffold-based tissues (myobundles) [57, 93, 159, 232]. Myosphere generation is based on the capacity of myoblasts to secrete their own extracellular matrix and self-organize into a 3D structure. These structures are cultured in free-floating state. However, myofibers in a myosphere lack the uniform alignment found in adult skeletal muscle [48]. Myobundles are mainly generated by embedding myoblasts in natural hydrogels (collagen or fibrin) to mimic extracellular matrix and create a biomimetic muscle microenvironment. Hydrogels are then casted between two fixed anchor points to maintain tissue under tension and allow unidirectional myofiber alignment when myoblast fusion is promoted [57, 65, 75, 93, 97].

### 3D skeletal muscle constructs

Several studies analyzed the metabolic characteristics of 3D skeletal muscle constructs and some have been compared to 2D myotubes. Glucose uptake and insulin sensitivity of human myobundles were explored [104]. Myobundles and 2D skeletal muscle cultures have comparable insulin-induced 2-deoxyglucose uptake but to a lesser than reported in vivo. However, myobundles better retain the insulin sensitivity of in vivo muscle than 2D monolayer cultures. These findings were expanded by a recent work showing that 3D structures prepared from muscle cells isolated from skeletal muscles of diabetic rats exhibited the metabolic characteristics of their native tissue [3]. Thus, myobundles prepared with muscle cells from healthy rats had a higher insulin-stimulated glucose uptake response than myobundles from diabetic rats. Glucose and oleic acid uptake and oxidation of 3D primary human myospheres were also explored [53]. Myospheres oxidized less glucose than 2D myotube model while the uptake of glucose was similar in both models. By contrast, myospheres showed lower oleic acid uptake and oxidation than 2D cultures.

Considerable advances have been made to produce 3D skeletal muscle models that mimic important features of skeletal muscles in vivo but the model lacks the multicellular complexity of the muscle tissue in vivo due to their single-lineage origin [48, 100]. This limitation has been addressed by including cells of others lineages, such as endothelial cells and neural progenitors [4, 75]. Skeletal muscle is one of the major organs affected by metabolic diseases, but it should not be considered as an isolated organ. Hence, skeletal muscle communicates with other affected organs such as the liver and adipose tissue via secreted factors [100, 151]. The use of multiorgan-on-a-chip devices that support inter-organ crosstalk is expected to provide promising insights into the understanding of pathophysiology of metabolic diseases without using living animals [69].

In conclusion, several in vitro models are available to mimic the response of the skeletal muscle to a pathological state or an intervention. The recent development of 3D models allows new perspectives that mimic better a physiological state, offering promising perspective in line of the 3R rules. However, the implementation of 3D structures could be complex to set up. More details about the advantages and limits of 3D models will be discussed later in this review. Depending on the objective, a 2D myotube system may be satisfactory.

## Adipose tissue-derived human mesenchymal stem cells

### Definition and characteristics of mesenchymal stem cells

The adipose tissue (AT) contains a heterogeneous mixture of cell types [105]. Approximately half of its composition is composed by mature adipocytes, while the rest is made up of a variety of cells, such as preadipocytes, endothelial cells, fibroblasts, monocytes, macrophages, lymphocytes, among others [35]. Although preadipocytes were initially believed to be the only adipocyte precursors, Zuk et al. discovered mesenchymal stem cells (MSCs) within AT, sparking interest in its therapeutic potential for cell-based treatments [231]. Adipose tissue-derived mesenchymal stem cells (ADMSCs) make up a significant proportion of the stromal vascular fraction, a component of adipose tissue that includes various cell types such as endothelial cells, immune cells, and stem cells [105].

Compared to other sources of MSC, such as bone marrow, adipose tissue yields a higher concentration of stem cells [231]. Like other MSCs, ADMSCs can differentiate into various mesodermal lineages, such as adipocytes, osteocytes, and chondrocytes [62]. These cells also exhibit self-renewal properties, allowing them to proliferate and maintain their population over time. ADMSCs express specific surface markers, such as CD105, CD73, and CD90, which aid in their identification, while they do not express hematopoietic markers like CD45 or CD34 [62].

### Cases of use in obesity-induced metabolic conditions

ADMSCs are increasingly recognized for their therapeutic potential due to their easy accessibility, abundance, and regenerative properties. One of the critical advantages of ADMSCs is their immunomodulatory capacity, which allows them to influence the immune response [167]. They secrete paracrine factors that can modulate the activation and proliferation of immune cells, including T cells, macrophages, and dendritic cells. This makes them particularly useful in studying inflammatory and autoimmune diseases like multiple sclerosis and Crohn’s disease [211].

ADMSCs have been studied extensively in both animal models and human clinical trials. In obesity, ADMSCs have been shown to reduce body weight and adipocyte hypertrophy, contributing to better metabolic health (reviewed in [121]). For example, experiments in high-fat diet-induced obese mice demonstrated that the systemic administration of ADMSCs reduced body fat mass and improved insulin sensitivity [30, 179]. Similarly, ADMSCs reduced body weight and visceral fat accumulation in leptin receptor-deficient mice [119], highlighting their potential as a therapeutic tool for obesity management.

In T2DM, ADMSCs have improved glucose homeostasis and insulin sensitivity. Studies have shown that ADMSCs can upregulate GLUT4 expression in adipose tissue and skeletal muscle, promoting glucose uptake and reducing blood glucose levels [108]. In addition, ADMSCs can differentiate into insulin-producing cells [54, 96, 199], offering a novel approach to restore pancreatic function in diabetic patients.

In liver diseases, ADMSCs have shown potential in treating non-alcoholic fatty liver disease (NAFLD) and its more severe form, non-alcoholic steatohepatitis (NASH) (reviewed in [119]). Animal studies have demonstrated that ADMSCs can reduce hepatic inflammation, promote liver regeneration, and improve liver function [68, 115, 170]. ADMSC-derived hepatocyte-like cells have been transplanted into models of liver injury, resulting in reduced fibrosis and improved liver function [13, 215].

ADMSCs have also been explored in treating chronic wounds and ischemic conditions. Their ability to promote angiogenesis and tissue repair makes them suitable candidates for treating *non-healing wounds*, such as diabetic ulcers and venous leg ulcers [83, 206]. ADMSC-based therapies have also been studied in myocardial infarction models, where they have been shown to improve cardiac function and reduce area of infarction by generating cardiomyocyte-like cells that integrate into the damaged heart tissue [11].

Beyond their therapeutic applications, ADMSCs can also serve as a platform for testing and screening bioactive compounds. Their human origin, responsiveness to metabolic and inflammatory stimuli, and capacity to differentiate into multiple lineages make them a relevant model to assess compound effects on adipogenesis, inflammation, and insulin sensitivity in vitro [90, 209].

### Advantages and limitations

One of the primary advantages of ADMSCs is their abundance and ease of isolation. Adipose tissue is more accessible than other sources of MSCs, such as bone marrow, and yields a higher number of stem cells. This makes ADMSCs a more practical option for large-scale clinical applications. Additionally, ADMSCs possess potent immunomodulatory properties, which allow them to suppress immune responses and promote tissue healing. This feature is particularly beneficial in treating autoimmune and inflammatory diseases.

ADMSCs can be genetically modified to improve their survival, ability to home to inflammation sites, and immunomodulatory properties. This increases their versatility in regenerative medicine and boosts their therapeutic potential (reviewed in [121]).

Despite these advantages, there are several limitations to the use of ADMSCs in clinical settings. One major limitation is the variability in the quality and function of ADMSCs depending on the source of adipose tissue. For instance, ADMSCs derived from subcutaneous adipose tissue have greater adipogenic differentiation capacity than those from visceral adipose tissue [203]. This variability can affect the consistency and efficacy of ADMSC-based therapies.

Another limitation is the potential for tumorigenicity. Although ADMSCs are generally considered safe, there is a concern that their prolonged proliferation and differentiation could lead to the formation of tumours [107]. This risk must be carefully monitored in clinical trials. Additionally, the long-term effects of ADMSC-based therapies are still not fully understood, and further research is needed to determine their safety and efficacy in human patients.

Furthermore, many bioactive compounds used to stimulate or modulate ADMSC activity in vitro may not reflect the actual metabolites reaching target tissues in vivo, especially in the case of dietary polyphenols and other complex molecules. This discrepancy should be taken into account when interpreting the translational potential of in vitro findings.

Finally, the scalability of ADMSC-based therapies presents a challenge. While ADMSCs can be easily expanded ex vivo, maintaining their differentiation potential and immunomodulatory properties during large-scale production can be difficult. Ensuring the quality and consistency of ADMSCs for therapeutic use will require standardized protocols and rigorous quality control measures.

In conclusion, ADMSCs offer significant potential for treating a wide range of diseases, from metabolic disorders such as obesity and diabetes to liver diseases and tissue repair. Their abundance, easy isolation, and regenerative properties make them a valuable tool in regenerative medicine. However, there are still challenges to overcome, including variability in cell function, potential safety concerns, and the need for standardized production methods. Despite these limitations, ADMSCs represent a promising avenue for future therapeutic developments, with ongoing research aimed at unlocking their full potential in clinical applications.

## Preclinical studies with human adipose tissue explants

### Advantages of using human adipose tissue explants

White adipose tissue (WAT) in humans is heterogeneous, highly vascularized, and innervated connective tissue. In addition to storing energy, it plays a key role in metabolic and endocrine communication with organs such as the liver, skeletal muscle, and brain [200]. Its function is regulated by both short- and long-term mechanisms, influenced by hormonal, nutritional, and developmental factors. The choice between using whole adipose tissue or isolated adipocytes in experimental protocols depends on the research objective: primary adipocytes are useful for acute metabolic studies but not suitable for long-term experiments.

WAT explants are an experimental technique initiated in the late 1960 s by Slavin and Elias [184], although it was Smith who proposed their use for long-term studies [185]. Thus, they constitute a valuable tool in biomedical research because they preserve the architecture and physiological function of the tissue in vivo. Explants preserve the cellular heterogeneity and paracrine signals of this tissue by maintaining the existing cross-communication between the different cell types, allowing the investigation of secretory products and their regulation in a physiological microenvironment. This model provides the native extracellular matrix (ECM), cell-cell interactions, and 3D structure, closely mimicking in vivo conditions compared to 2D cell models. Human WAT explants retain multiple cell types, such as adipocytes, stromal vascular fraction (SVF), preadipocytes, immune cells, and endothelial cells, facilitating research on inflammation, angiogenesis, and tissue remodelling. So, it makes possible to study cellular interactions and biological processes in an environment that reflects the complexity of adipose tissue compared to cells isolated in culture. Other techniques in which it is necessary to separate and purify the cells require treatment with collagenase and this may involve some physiological modification in the cell structure or artificially activate the cells [197].

WAT explants can be used to evaluate bioactive compounds and their potential interactions with hormones such as leptin, adiponectin, and inflammatory cytokines. They allow for the analysis of processes like lipolysis, lipid uptake, and glucose metabolism in a near-natural environment. At the same time, explants enable the assessment of drugs, translational research, and other interventions directly on human tissue, reducing the gap between preclinical and clinical research. This model brings us closer to human physiology compared to animal models [35]. Additionally, they provide an ethical alternative to animal testing, addressing concerns about species-specific metabolic differences.

WAT is readily available as a by-product of surgical procedures like liposuction, abdominoplasty and bariatric surgery, making it an ethically less controversial source. Although the collection of adipose tissue samples from humans is relatively easy to process and culture compared to other human tissues, it must comply with specific criteria and requires strict ethical compliance.

WAT explants can remain viable for days to weeks under appropriate culture conditions, allowing for extended observation of dynamic processes with bioactive compounds or drug responses. Another advantage is that it allows the human personalized research, including examining differences based on age, sex, body mass index (BMI), health conditions and comparing subcutaneous and visceral fat depots.

### Limitations and challenges associated with adipose tissue explants

While human adipose tissue explants offer significant advantages, there are also several limitations associated with their use. Among these disadvantages, one of the most significant is the variability in the samples. There can be important differences in age, sex, BMI, genetic background, and health conditions of donors, which introduce heterogeneity in the results. In addition, variability in the size and quality of the tissue sample affects reproducibility between experiments. Also, subcutaneous and visceral fat depots differ metabolically and structurally, which complicates the interpretation of the results, and differences in how the tissue is collected and processed (e.g., liposuction vs. surgical excision) can affect the quality and consistency of the sample [35]. On the other hand, while explants maintain local interactions between cells, they lack systemic input from other tissues, such as hormonal regulation and neuronal input, limiting their ability to replicate whole-body responses.

Although they can survive for days or weeks in culture, their viability and functionality decline over time [168]. Maintaining the viability of multiple cell types within explants requires precise control of culture media, oxygen levels, and other factors. Prolonged culture can cause dedifferentiation of adipocytes, which alters their metabolic and functional properties [77].

Another limitation of this technique is that there may be a lack of control over cell types. Being a heterogeneous cell population containing a mixture of adipocytes, immune cells, endothelial cells, and others, it is more difficult to extrapolate the effects of the individual compounds tested on the different cell types that make up the sample. Crosstalk between different cell types can complicate data interpretation, particularly in studies focusing on specific signalling pathways. Adipose explants are not readily amenable to gene editing or other experimental manipulations that are straightforward in cell lines or animal models. Culture of human adipose tissue explants leads to profound alterations of adipocyte gene expression [73].

As described above, obtaining adipose tissue requires strict ethical compliance and some donors may not consent to the use of their tissue for research purposes. Each experimental protocol must have been previously approved by the Clinical Research Ethics Committee, in accordance with the provisions of the regulations on the treatment of biological samples of human origin, as well as the Protection of Personal Data and guarantee of digital rights.

Briefly, despite the limitations offered by this methodology, the human adipose tissue explants are a valuable research tool due to their ability to maintain physiological relevance and provide versatility in the study of bioactive compounds.

## Spheroids and organoids

Given the growing interest in the use of 3D cell culture models, it is essential to provide an overview of the advantages and limitations of these approaches. Particularly, spheroids and organoids are structures composed of multiple cells that are been used in the last years to mimic the physiological conditions found in the original tissues.

## Spheroids

### Definition of spheroids

Another type of model that allows the study of mechanism of action of bioactive compounds are spheroids. Spheroids are a type of 3D culture (cellular aggregates) that simulate a specific cell function and morphology. Spheroids replicate more accurately the extracellular environment in vivo as compared to standard 2D cell cultures, including cell-to-cell and cell-matrix interactions and cell signaling pathways [41]. In this model, suspension cells adhere/aggregate to form a rounded (spheroid) shape in culture (in vitro) which can predict in vivo responses and thus, is useful for pre-clinical studies with therapeutic approaches (such as drug screening, transplantations) [163]. Moreover, they are also allowing basic studies on cell and tissue function in a more comprehensive manner.

There are several methods to develop spheroids such as hanging drops, gel embedding, scaffolds, magnetic levitation, and spinner culture [163]. To study spheroid cell culture, various kinds of biomaterials are used as building forms of hydrogel, film, particle, and bead, depending upon the requirement. In addition, studies should focus on methods to dissociate cells from spheroid into single cells. Applied materials and methods vary greatly and are reliant on spheroid specific cell type and in vivo tissue architecture (reviewed in [114]).

### Advantages and indications

Spheroids display important advantages including functional versatility. Spheroids can be cultured in combination with a wide range of conditions and co-adjuvants mimicking particular metabolic environment/conditions. For instance, gut microbiota is considered a key factor in modulating metabolic health. Indeed, bacterial-origin bioactive compounds are considered to be important metabolites in modulating metabolic health [155]. A novel protocol of 3D models selectively grows bacteria within the core of tumour spheroids which has allowed their continuous and parallel profiling in physiologically relevant conditions. Tumour spheroids are incubated with bacteria in a low-adhesion plate followed by washing steps and an antibiotic selection protocol. This confine bacterial growth within hypoxic and necrotic core of tumour spheroids [85]. This opens the door for spheroids culture in combination with specific bacterial species allowing the simulation of a customized spheroid-gut environment, which could be of interest in metabolic diseases. Importantly, small methodological variations might allow the study of bacterial origin compounds of biological interest in (human) spheroids. Another interesting condition is the co-culture with endothelial cells allowing the formation of vessel-like structures [154].

Given the already described biological advantages, spheroids could be developed in murine and human cell types. Development of human spheroids allows more translatability of research outcomes as compared to non-human (murine) models. However, specific microenvironment conditions should be carefully selected to be aligned as much as possible to the physiological *milieu*. Indeed, human origin spheroids might represent an up-grade level of conventional 2D cell culture. These allow adaptability of specific cell types in a nutrient and therapeutically-modified environment accordingly to particular experimental setup or clinical context [156]. Providing all the above, among the outstanding indications of spheroids models are their suitability for drug testing [210] and prediction of treatment response (eventual biomarkers) [204]. Obtained results in this line are very promising and spheroids could be used to better understand the physiological cues of cancer and metabolic diseases as well as their development, progression and therapeutic effectiveness.

Nevertheless, spheroids also have some disadvantages (caveats). In technical terms, spheroids pose limitations such as cell death induced by hypoxia and necrosis in the spheroid core [163]. Additionally, if evaluating physiological effects of bioactive compounds, spheroids architecture might difficult an equimolar distribution of bioactive/chemical compounds (including nutrients and metabolites) in the global surface and core of the spheroid structure, with subsequent changes in osmolarity [21].

### Comparison to animal/human studies

Spheroids or 3D cultures seem an interesting tool to overcome limitations of traditional 2D cell cultures which do not represent the physiological *milieu* to a full extent. Retaining tissue microenvironment with 3D cultures is a major advance to display more biological complexity in a particular experimental setting while still maintaining in vitro advantages such as simplicity and cost-efficiency. However, spheroids cannot simulate the overall in vivo context and other biological interactions (tissue-tissue or inter-organ communication) are missing. It could be stated that spheroids represent a middle-edge between 2D cell cultures (in vitro) and animal (in vivo) models, allowing the most physiological shape of cells of interest and cell-to-cell contacts [133]. Nevertheless, spheroids constitute a more translational approach vs. traditional 2D cultures, particularly, of human origin, which warrants their applicability and translational potential.

### Relevant examples: types and models

Spheroid models have been described within the field of metabolic diseases; of particular interest are those of human origin. Among the most widely used, we find tumour spheroids such as hepatoblastoma spheroid cultures (derived from patients). This metabolically relevant model can predict differential responses to chemotherapy treatment allowing a step forward to personalized precision medicine [186]. Other spheroids cultures include culture of human hepatocytes, cardiomyocytes or even neuronal spheroids [14, 44, 49, 158]. They maintain their functional profile in terms of transcriptomic, metabolomic, proteomic features [141], lipidomic profile [173], and lifespan [16] for an extensive time-frame (weeks). Indeed, hepatocyte spheroids allow the study of drug-induced effects on liver, similarly as bioactive compounds with interest in metabolic diseases [19].

One of the most relevant cell types in the field of metabolic diseases are adipose spheroids. Specifically, human adipose spheroids represent better adipocyte morphology and function in vivo, similarly as in fat depots [64], as compared to standard 2D adipocyte cell cultures. Some approaches have been already described for differentiation of human adipose spheroids culture which overcome some limitations of 2D cultures. These adipocyte spheroids not only retain morpho-functional features of mature (in vivo) adipocytes but also allow the possibility to add other cell types present in adipose tissue (i.e. endothelial cells) towards development of complex adipose tissue organoids [91]. Adipose spheroids retain adipogenesis properties and feature cellular and molecular phenotypes as mature adipocytes. They constitute a suitable model to evaluate nutritional challenges such as FFA supplementation without altering their viability and showing positive control responses against these perturbations [173]. Adipose spheroids represent one of the most promising 3D models to evaluate the effects of bioactive compounds in the field of metabolic diseases.

Bioactive compounds such as polyphenols have beneficial effects on health and metabolism, including antioxidant, anti-glycemic, anti-obesity, anti-inflammatory properties. However, development of physiologically relevant methods based on cell-cell interactions are needed to deeply explore and characterize these biological actions of these compounds [80]. Indeed, newly published articles have shown that secondary metabolites (from sand-dune or *Humulus lupulus*, for instance) induce cytotoxic actions in cancer (breast and colorectal) spheroid cell lines [17, 74]. Similarly, Vargová et al. reported the ability of hypericin, a bioactive compound of St. John’s Wort (*Hypericum perforatum*), to reversibly decrease site population proportion, a *bona fide* cancer stem-like cell marker, through competitive inhibition of ABC transporter BCRP, which may account for multidrug resistance [207]. Further evidence has shown that cancer spheroids allow evaluation of antioxidant and anti-proliferative potential of seed extracts with high content of polyphenols from medicinal plants [70] as well as glucose uptake and lipid content (among other activities) from marine seagrass metabolites, some of them, with already reported activities in metabolic syndrome, such as cirsimarin or spiraeoside [18].

Notably, most studies carried out so far have been applied and developed mostly in cancer models. However, evidence of application of spheroids in the effects of bioactive compounds in metabolic diseases is still very limited although relevant [18]. Only few studies have investigated the effects of some dietary fatty acids but only within a characterization context rather than bioactive compounds screening [173]. Evaluation of anti-obesity and/or anti-diabetic properties of novel bioactive compounds in adipose spheroids will undoubtedly drive a major trend in metabolic field in coming years. Particularly, regarding the processes of whitening and browning of adipocytes that are closely associated with development of metabolic disorders, it has been reported that brite/beige/brown adipocytes can be maintained in 3D cultures and that can respond to different drugs [64, 101, 158].

### Application: present and future research

Extended use of spheroids models in metabolic diseases context will contribute to reduce the number of studies performed in mammals and will avoid related ethical concerns. Spheroids provide a more resemblance to in vivo conditions. Thus, spheroids models are of preferable choice to address physiological effects of bioactive compounds. Spheroids enable the study of cell-cell interactions and more complex environments which also allow basic studies on cell and tissue function.

Given that spheroids (of human origin, particularly) represent a more translational model, a short time lapse between experimental discovery and (clinical) application is warranted and these models could contribute to pave the way towards a personalized and precision medicine. However, further studies are needed to get new insights about application of spheroids and 3D models in basic and clinical research. In summary, spheroids are an interesting alternative for future studies of bioactive compounds on metabolic diseases mainly due to their translation and versatility.

## Organoids

### Concept and characteristics of organoids

Organoids are 3D organ-like or tissue structures grown in vitro. Organoids consist of various cell types that are able to self-organize and recapitulate the key aspects of the structure and functionality of the derived organ [218]. The term organoid was first coined in 1946 to describe a cystic teratoma grown in a 2-month-old infant; however, it was not until 2008 that the development of stem cell culture techniques allowed the establishment of organoids as we know them today [94].

Organoid cultures enable a more accurate replication of physiology, which cannot be achieved with conventional 2D cultures, nor with non-primate animal models [125]. In this sense, organoids have been postulated as an alternative approach to reduce the gap between the traditional in vitro 2D cultures and in vivo assays. 2D cell cultures are easier to maintain and cost-effective, but they lack the complexity and interactions of in vivo systems. However, the absence of cell-cell and cell-extracellular environment interactions limits cell differentiation, proliferation, gene and protein expression, and responsiveness to stimuli. Cell morphology and division are also affected as a result of 2D culturing, impacting cell phenotype and function [95, 218].

Organoids are developed from stem cells or organ-specific progenitors, such as pluripotent stem cells (PSCs) including embryonic stem cells (ESCs) and induced PSCs (iPSCs), or adult stem cells (ASCs). Tumor tissues from patients can also be used to establish cancer-derived organoids, called tumoroids. Each of these cell types is capable of reaching a certain level of maturation, which restricts the application of the organoid. In this line, PSC-derived organoids, also called “embryoids”, do not achieve complete maturation, and instead they exhibit characteristics similar to foetal tissues. ESCs-derived organoids present higher maturation than iPSCs, which make them more suitable for studying tissue development at later stages of organogenesis. On the other hand, iPSCs can be used as models for early phases of organogenesis [218]. Nevertheless, it is important to consider that ESCs are derived from embryonic tissue, which involves ethical issues. An interesting feature of embryonic organoids is the coexistence of cells from the different germ layers, unlike in classic organoids, which are limited to cell subsets from one germ layer [161].

By contrast, ASCs more closely resemble adult tissues, due to their ability to fully differentiate and thus, they are more suitable to study issues related to adult tissue biology [161].

### Usefulness of organoids

Organoids provide a unique tool for disease modeling. Since organoids mimic the features of their tissue of origin and thus, replicate pathological traits, the development of organoids harbouring a particular mutation that causes a certain disease, serves to study the underlying mechanisms of organ-specific genetic diseases. For example, cystic fibrosis organoid models or retinal organoids models have been established from humans and rodents [216, 218]. Similarly, organoids can also be used to study infectious diseases allowing the study of the interaction between these microorganisms such us bacteria, viruses or protozoa, and their host cells. In this line, for example, it has been possible to culture liver organoids infected by hepatitis B, C and E viruses, and bronchial organoids to mimic SARS-CoV-2 infection. There are also organoid models available for modelling certain metabolic diseases such as obesity, as well as alcoholic and non-alcoholic fatty liver disease. Moreover, this innovative technology allows the development of models for cancer research that more faithfully capture the genetic heterogeneity of the original tumour compared to primary tumour cell culture, while also mirroring the features of the patient’s tumour [218].

Beyond disease modelling, organoids also serve as a tool for drug screening and development as well as for evaluating drug toxicity and safety. In particular, using patient-derived organoids it becomes possible to predict the response of an individual patient to a specific compound or drug, allowing the development of personalised treatments, which has led to significant progress in the field of precision medicine. Finally, tissue engineering and regenerative medicine have also witnessed significant advances using organoids to repair and regenerate tissues. What is more, organoid technology has emerged as promising tool for autologous transplantation, thereby reducing the risk of immunological rejection. Preliminary experiments conducted in animal models have demonstrated the feasibility of this approach. For instance, retinal sheets obtained from mouse ESCs and iPSCs have been successfully transplanted into a mouse model with retinal degeneration. In the case of humans, patient-derived intestinal stem cells that underwent genetic editing to generate functional organoids have been transplanted into individuals with cystic fibrosis [216, 218].

### Needs and limitations

Organoid culture requires the recreation of the microenvironment, also referred as stem cell niche, necessary for the correct self-organised growth and differentiation of the organoids, which is initiated by specific soluble biochemical signals found within the niche [125]. This niche can be achieved by cultivating the organoids embedded in extracellular matrix (ECM) gels that provide support for cell growth. Matrigel, the most common matrix for 3D culture, is an animal derived hydrogel which contains all the components and growth factors to promote an efficient cell growth and differentiation [161]. However, its composition can vary from one batch to another, it can induce immune system response, and it can impede the infiltration of exogenous substances. Therefore, chemically defined synthetic hydrogels have been developed, tailored to the organoid’s needs and thus, allowing the control of culture environment [218]. Other approaches to establish organoids consist on culturing cell aggregates in suspension or by air-liquid-interface (ALI), another method where the basal cell layers grown in contact with the culture medium, while the external layers are exposed to the air allowing a higher oxygen supply than submerged organoid cultures [161].

Despite the significant advances that the culture and use of organoids has brought to many fields, there are still some challenges that need to be addressed. For example, although current organoid systems can achieve a significant degree of functionality, they still fail to replicate all the functions found in an organ. This is in part due to their limited lifespan, which restrains the ability to develop to a fully mature stage and thus, to differentiate into the full set of differentiated cell types found in vivo (this is specially the case for PSC-derived organoids). On the other hand, as organoids grow, nutrient accessibility and waste removal decrease, leading to necrosis and as consequence, to a shorter lifespan. In some cases, this issue can be solved by fragmenting and reseeding the organoids or using bioreactors. In other cases, the co-culture of organoids with endothelial cells has shown to successfully promote vascularization, enabling the transport of nutrients and waste, although it is very challenging to establish technically. Other important aspect to highlight is that nowadays the production of organoids is limited to a few laboratories. This is primarily due to the high cost associated with growth factors and additives added into the culture medium [218]. Additionally, the absence of standardized protocols that limit reproducibility and the difficulty of monitoring cells within the 3D culture further contribute to the limited availability of organoid cultures [180].

Although it is still an emerging technology, 3D bioprinting of organoids has emerged as a new opportunity to continue with advances in the development of organoid systems. With this technology, undifferentiated or differentiated stem cells or even the organoids themselves can be bioprinted, allowing opportunity to more precisely control spatial configuration, to facilitate fusion in order to create larger tissues in vitro, as well as allowing a more precise adjustment of the biochemical and biophysical signals that facilitate the growth [125].

Despite the current drawbacks and that it is still an emerging field, organoid technology has demonstrated the potential to transform basic and clinical research, while serving as an alternative to in vivo experimental models.

**Fig. 1:**
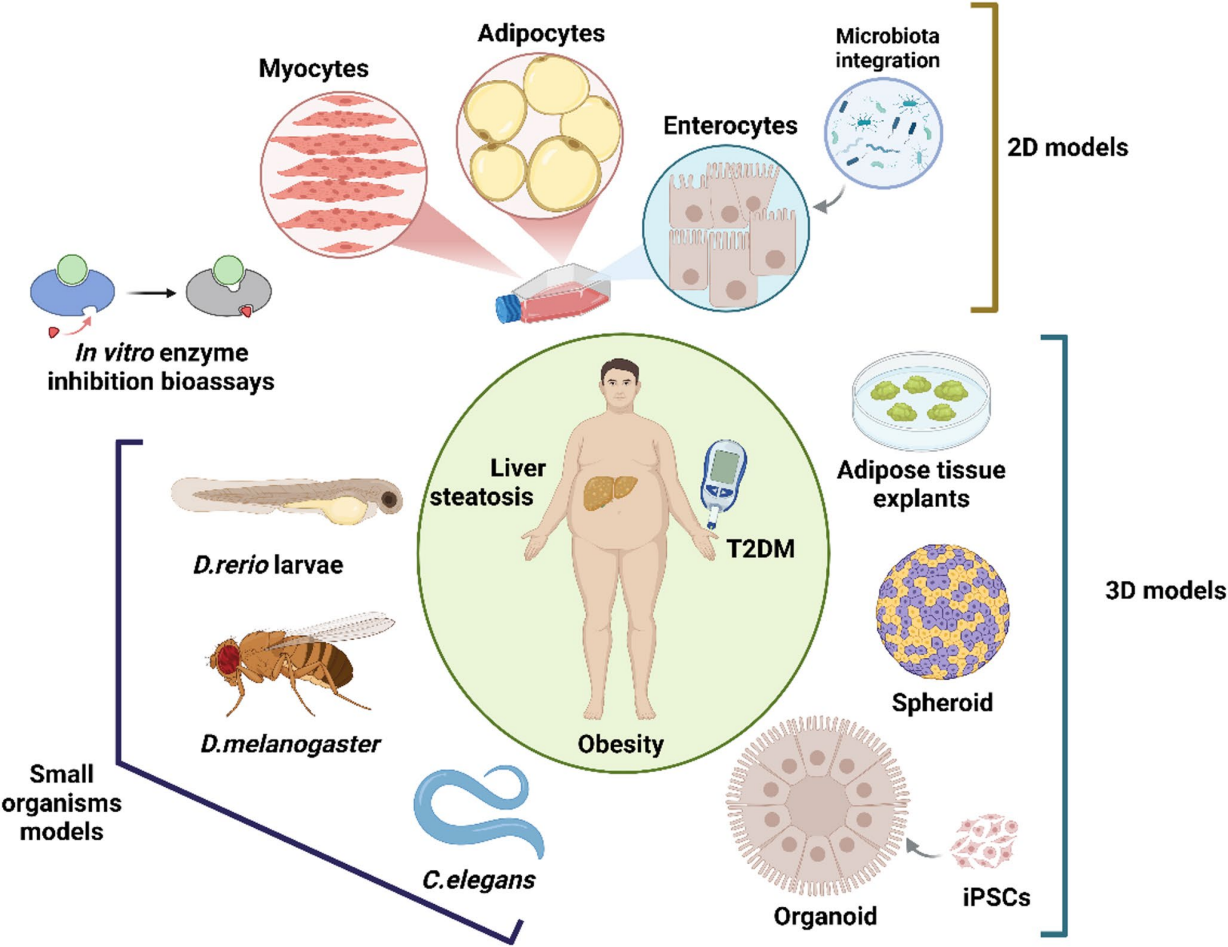
Representative image of the most important pre-clinical models applied to study metabolic diseases and that are explained in detail in the present review. These methods include in vitro enzyme assays, cell culture (skeletal muscle, enterocytes/intestinal cells, human adipose mesenchymal stem cells) three-dimensional cultures (spheroids, organoids) and small (non-mammalian) organisms. Two different cell types can be combined in 2D cell co-cultures to study cell-cell interactions. Small organisms such as *C. elegans*, *D. rerio* and *D. melanogaster* offer a straight-forward approach to assess in vivo effects in an effective, time-wise manner while avoiding major ethical concerns. Image created with BioRender (https://www.biorender.com/)

Figure 1 summarizes the most relevant pre-clinical models traditionally used in pre-clinical research related to obesity and metabolic diseases.

## Small organisms

### *Caenorhabditis elegans*

*Caenorhabditis elegans* (*C. elegans*) is a microscopic, free-living nematode (roundworm) that has been extensively employed as an animal model organism in different diseases and physiological processes, including diabetes, obesity, aging, cancer and neurodegenerative disorders [174, 175].

Its simple structure and transparency make it ideal for genetic, developmental, and neurobiological studies. Other advantages that make *C. elegans* a very convenient research model are its small size (1 mm in length and 0.1 mm in width), the large number of progenies, the short life span (it takes around 72 h to reach adulthood), and its completely sequenced genome [135]. Approximately 99.9% of individuals are hermaphrodites, capable of self-fertilization, while around 0.1% are males. *C. elegans* primarily feeds on bacteria, being *Escherichia coli* (*E. coli*) the most commonly used food source [175].

The nematode structure is composed by three regions: head, which contains sensory structures, the terminal mouth (stoma) and the muscular pharynx; the body, covered by a rigid cuticle that protects the organism; and the tail, which contains the anus at the posterior end. This tail differs in male worms, with a specialized structure called spicules used during mating. In this simple structure, however, *C. elegans* possesses a digestive system, which comprises the mouth, pharynx, intestine, and anus, a simple nervous system composed by 302 neurons, a muscle system, which enables the movement of the worm, a reproductive system (hermaphrodites have both male and female reproductive organs, while males possess only male structures), and an excretory system, composed by a single excretory cell with a tubular structure [174].

When cultured at 20 °C, *C. elegans* progresses through several distinct stages during its life cycle, starting from an egg stage to an L4 adulthood. After approximately 14 h of embryonic development, the egg hatches into the L1 larval stage, where the worm begins feeding and growing. Following the first molt the worm enters the L2 stage, characterized by continued development of its digestive and reproductive systems. Under adverse conditions, the L2 larvae can enter the *dauer* stage, a stress-resistant, non-aging form that allows survival until conditions improve. Otherwise, the worm progresses to the L3 stage, where it undergoes further growth. After another molt the worm enters the L4 stage, where reproductive organs fully develop, particularly in hermaphrodites. Finally, the worm matures into an L4 adult capable of reproduction, with a lifespan of 15–21 days. All these characteristics allow to culture and manipulate this nematode very easily at low cost through conventional in vitro methods [5, 118].

*C. elegans* is widely known for being the first animal model whose genome was completely sequenced [29]. Despite the evolutive difference, *C. elegans* shares about 60–80% of its genes with humans, including key genes involved in lipid metabolism and energy homeostasis (intestinal function, fat metabolism and appetite), which are particularly well conserved [42, 67].

Interestingly, *C. elegans* genome can be manipulated genetically with numerous tools, such as CRISPR-Cas9 gene editing and RNA interference (RNAi). Mutant nematodes with reporter genes aid in gene expression assessment, and low-cost mutant strains are accessible from the *Caenorhabditis Genetic Center* (National Institutes of Health (NIH)). In addition, *C. elegans* use for experiments does not require the approval of the Institutional Animal Care and Use Committee [133]. For these reasons, this in vivo laboratory model has emerged as an experimental model for the study of the pathogenesis of different diseases, especially in those that affect lipid and carbohydrate metabolism, fat accumulation and health span [42, 67].

### *C. elegans* as a model for obesity research

The identification of functional ingredients and/or bioactive compounds with potential activity in regulating energy homeostasis represent a potential tool for the management and prevention of obesity-related diseases [146]. In this context, the use of multicellular in vivo models allows us to understand the metabolic effects of these ingredients and to elucidate the specific mechanisms of action involved in this regulation.

*C. elegans* has been demonstrated to constitute a powerful model for exploring the genetic basis of fatty acid synthesis and the regulation of fat storage [213]. In *C. elegans*, more than 400 genes involved in fat storage are evolutionarily conserved with mammals [174]. As previously mentioned, *C. elegans* mainly obtain fatty acids from the bacteria diet (most commonly *E. coli* OP50 strain) through digestion or an endogenous *de novo* synthesis in the cytoplasm. Initially, fatty acid synthesis is started by acetyl-CoA carboxylase (ACC/POD-2), which transforms acetyl-CoA in malonyl-CoA, which is then elongated by fatty acid synthase (FAS/FASN-1), to generate palmitic acid. Saturated palmitic acid can be used to constitute TAGs or phospholipids, but also can be modified by fatty acid elongases (encoded by *elo* genes) and desaturases (encoded by *fat* genes) in the biosynthesis of various polyunsaturated fatty acids (PUFAs) [5, 118]. Many key transcriptional regulators are involved in the modulation of the lipogenesis, desaturation and fatty acid oxidation. Thus, CEBP-2, the homolog of human CCAAT/enhancer-binding proteins, positively regulates acyl-CoA synthetase (ACS-2) and enoyl-CoA hydratase (ECH- 1). SBP-1, the homolog of a sterol regulatory element-binding protein in mammals, positively regulates fatty acid desaturases (FAT-6/7), fatty acid synthase (FASN-1), and polarity and osmotic sensitivity defect (POD-2, homolog of acetyl-CoA carboxylase), modulating lipogenesis. On the contrary, AAK-2, the homolog of mammal AMP-activated protein kinase, positively regulates adipose triglyceride lipase (ATGL-1), which, together with hormone-sensitive lipase (HSL), are key lipases capable of hydrolysing acyl esters, inducing lipolysis [5]. Moreover, numerous acyl-CoA synthases (ACS), dehydrogenases (ACD), enoyl-CoA hydratases (ECH), and thiolases play important role in the lipid catabolism through the mitochondrial and peroxisomal β-oxidation pathways. Finally, another key protein in *C. elegans* is NHR-49, the homolog of mammal peroxisome proliferator-activated receptors, which modulates the expression of fat synthesis genes, and beta-oxidation genes [5, 118].

As *C. elegans* is a simple organism, its lipid metabolism is connected and related to many other factors, including oxidative stress, ageing and longevity. Indeed, several studies have found a relationship between glucose metabolism operation and mitochondria function, dietary carbohydrate intake, fat accumulation and lipid metabolism operation [219]. Moreover, this nematode represents a reliable model to evaluate the effects of high carbohydrate diets (high-glucose or high-sucrose), trying to mimic overfeeding and diabetic conditions, which not only increase the fat storage, but also has an effect on oxidative stress, aging, and lifespan. In this context, it has been established that a shortened lifespan in high glucose-exposed worms is in part due to the activation of the insulin/IGF-1 signalling (IIS) pathway [133, 219].

Diverse mutants are available on the basis of wild-type nematodes because of their genetically tractable systems and the use of powerful genetic tools [175, 224]:

- *C. elegans* mutants with altered the insulin/IGF-1 signalling pathway (IIS), a crucial and well conserved signalling pathway in this nematode, where the *daf-2* gene is homologous to the insulin receptor in humans. Mutations in *daf-2* lead to changes in fat storage, providing a model of obesity linked to insulin resistance, a condition similar to T2DM in humans. These mutants serve as valuable tools for testing the effects of pharmacological interventions that might restore normal fat metabolism.
- Mutations in AMP-activated protein kinase (AMPK) signalling pathway, involved in regulating energy balance. Mutations in the *aak-2* gene mimics the effects of high-calorie diets in mammals, providing an insight into the cellular energy regulation and its disruption during obesity.
- Mutations in the mTOR signalling pathway, an important signalling pathway in the nutrient sensing and in the modulation of fat accumulation.
- Sirtuins (*sir-2.1*), involved in lifespan extension and metabolism, also play a role in fat storage, allowing researchers to study the interaction between aging, metabolism, and obesity.

### *C. elegans* as a model for screening bioactive ingredients with lipid-reducing activity

It is generally considered that *C. elegans* constitutes a convenient in vivo model for the screening of the effects of nutritional perturbations and the fatty acid modulation activity of bioactive components of the diet [174]. Although this nematode lacks the adipocytes present in mammalian adipose tissue, *C. elegans* stores fat in lipid droplets in its intestinal and hypodermal cells. These droplets are fat-storing organelles consisting of a hydrophobic core of TAG and cholesterol ester surrounded by a phospholipid monolayer containing several proteins [178].

Due to the transparency of the worm, the fat deposition of these lipid droplets can be quantified by different experimental tools, including label-free visual imaging, biochemical and chemical assays, and staining analysis of lipids. Thus, the lipid content can be easily visualized under microscopy by fat-soluble dyes, where Sudan Black B, BODIPY, Nile Red and Oil red O are the most common [5]. Among them, the quantification of the fluorescent fixative Nile Red lipophilic dye has been demonstrated to be a reliable method to determine the lipid content in this nematode [7, 133]. Additionally, due to its genetic plasticity, GFP-tagged lipid droplets (LD)-specific markers have been widely used to evaluate the effect of different bioactive compounds on the *C. elegans* lipid metabolism. Among these LD-markers, the short-chain dehydrogenase DHS-3 have been shown to be very enriched on the surface of the lipid droplets, so its fusion with GFP represents a reliable tool to investigate the regulatory mechanisms involved in the storage of fat [5].

Thus, *C. elegans* has been successfully implemented in different studies focused on the identification, targeting, screening and selection of different lipid-lowering compounds, including polyphenols, fatty acids, dietary fibres, probiotics, prebiotics and post-biotics, together with a large number of symbiotic combinations, and to improve the knowledge of their biomolecular mechanism of action [78]. Some examples are:

- **Polyphenols**: Different studies have reported the activity of some flavonoids and phenolic acids that, either supplemented as extracts or as single compounds, prolong *C. elegans* lifespan [81], exert antioxidant activities [10] and modulate lipid accumulation [7, 117]. Probably, the best studied polyphenol is resveratrol, which is able to modulate lipid metabolism [225], but also to extend lifespan and reduce oxidative stress by affecting the unfolded protein response of the endoplasmic reticulum (UPR^ER^), mimicking the response to calorie restriction [7].
- **Fatty acids**: Different MUFA and PUFA supplements have been shown to improve *C. elegans* health [133, 138], including lifespan extension [153]. Thus, oleic acid supplementation improves *C. elegans* lifespan by the activation of SKN-1, homologous to human Nrf1/NFE2L1 in the endoplasmic reticulum, which also affects to the lipid homeostasis [40]. In their study, Navarro-Herrera and colleagues reported that both omega-3 and omega-6 FAs induced fat-loss in *C. elegans*, being linoleic acid (LA), gamma-linolenic acid (GLA) and dihomo-gamma-linolenic acid (all of them omega-6) the most effective ones [138].
- **Probiotics and postbiotics**: During the last years, *C. elegans* has been demonstrated to represent a powerful method to investigate microorganism–host interactions, as well as to evaluate the antioxidant, anti-aging, and life-extending properties of different probiotics strains. *C. elegans* represents a reliable model to evaluate the potential anti-obesity, anti-diabetic, or anti-inflammatory properties of specific bacterial strains proposed as probiotics for the prevention of metabolic syndrome [78]. Thus, different *Bifidobacteria* and *Lactobacilli* strains have been demonstrated to extend nematode lifespan, mainly through modulations in the p-38 mitogen-activated protein kinases (p38-MAPK) signalling pathway [103].

### Limitations of the *C. elegans* model

Despite its advantages, *C. elegans* poses some limitations, including its simply physiology, which limits the understanding of complex biological systems, such as organ interactions and systemic responses, but also the lack of a proper immune system [133]. Although *C. elegans* exhibits some behavioural responses, such as chemotaxis and learning, its behavioural repertoire is limited compared to higher organisms, which represent a limitation in the study of complex behavioural diseases, such as those relevant to human neurodegenerative diseases or psychological disorders. Other limitations associated with its simplicity would be the greater simplicity of its genome, where there is not always a homologue for the entire mammalian genome, or its short life cycle, where the observed results regarding development are not always extrapolated to long-lived organisms.

In the case of lipid metabolism, the main limitation is attributed to *C. elegans* is that it lacks adipocytes, and the complete lipid profile remains unknown. Furthermore, this nematode is auxotroph for cholesterol [128]; although its genome contains some genes involved in cholesterol metabolism, *C. elegans* is not able to synthesize cholesterol [133]. However, despite its limitations, *C. elegans* represents a valuable and reliable research model in different diseases, especially in those related to lipid metabolism.

### *Danio rerio*

Zebrafish (*Danio rerio*) is an excellent model to study metabolic disorders because they possess each of the essential organs and regulatory mechanisms to control metabolism that humans have [34]. Obese zebrafish can be conveniently produced by overfeeding, starting from the onset of feeding at 5-day post-fertilization [226]. For zebrafish larvae and juveniles, chicken egg yolk solution is the most popular HF), though heavy cream has also been used [226]. Oka et al. reported the first adult diet-induced obese (DIO) zebrafish model, in which adult fish (3.5 months of age) were fed 60 mg or 5 mg of freshly hatched *Artemia* per day for 8 weeks (150 calories vs. 20 calories) [140]. Meguro et al. created another HFD for zebrafish that included 20% lard or maize oil [131]. In addition, zebrafish have been used to characterise genetic models of obesity. These models include mutants from targeted mutagenesis and genetic screens, as well as transgenic lines that express genes associated with obesity. For example, the *vizzini* mutant zebrafish has abnormally large subcutaneous lipid droplets even though the number of lipid droplets in adipocytes remains constant. A mutation in the growth hormone (GH) 1 gene (*gh1*) that results in an early stop codon is the cause of this fat distribution [130]. Models of obesity in transgenic zebrafish are frequently created by replicating current mammalian models. Similar to how it controls energy homeostasis in mammals, the central melanocortin system (CMS) in zebrafish consists of peptides derived from proopiomelanocortin (POMC), their receptors (MC3R and MC4R), and Agouti-related peptide (AgRP) [84]. Overexpressing AgRP [Tg(b-actin: AgRP)] in zebrafish has resulted in the development of a genetic model of obesity [187]. At all stages, the transgenic zebrafish show increased body weight, linear growth, visceral adipose accumulation, and total triglycerides (TG).

The zebrafish pancreas has been used to study the pathophysiology of T2DM because its exocrine and endocrine components are comparable to those of mammals and because it can regenerate [123]. Immersion in a 4% glucose solution for 5 days is an established model for inducing hyperglycaemia in larvae [182]. In the case of adults, immersion in 111 nM glucose for 14 days was able to increase glucose levels about 4-5-fold, however, the blood glucose level decreased after 7 days of glucose withdrawal, but it was still almost 2 times higher than the control group [33]. In another study, continual immersion of adults in a 2% glucose solution for 30 days induced transient hyperglycaemia and fluctuated depending on glucose levels in environment [76]. Gradational increasing of glucose concentration (from 1% → 2% → 3%) for 2 months resulted in hyperglycaemia that is persistent for 2 months in younger zebrafish adults [52]. Different experimental protocols have been developed to use the DIO method to induce T2DM in zebrafish. Authors reported that 6-fold overfeeding *per* day with otohime B2 containing 11% crude fat, 51% crude protein, 2.3% crude calcium, 1.5% phosphorous, a maximum of 15% ash, 3% crude fibre for 8 weeks induces significantly increased glucose levels from 46 ± 5 mg/dL to 68 ± 11 mg/dL, inducing hyperglycaemia in adult zebrafish [227]. Other authors induced insulin resistance in adult zebrafish fed twice daily with HFD (1% egg yolk) together with brine shrimp (60 mg cysts) [132]. The combination of DIO and glucose concentration method was first developed by Wang et al. [212]. They fed 10% cholesterol-rich food to zebrafish larvae while simultaneously exposing them to 2% glucose concentration. This model had advantages in that persistent hyperglycaemia was lacking in the glucose concentration method but was possible in this combined method. Likewise, the longer induction time in the DIO approach (usually 6–10 weeks) was greatly reduced to 2 weeks. The same method was applied to adult zebrafish with little modification by the same author using 3% glucose concentration and the same content of cholesterol in the diet for 19 days [208]. The result of this model resembled the phenotype of T2DM in humans. Transgenic models of insulin resistance haven been also developed. In one model, skeletal muscle insulin resistance was achieved by transgenic expression of a dominant-negative IGF-I receptor (IGF-IR) in skeletal muscle. The transgenic fish showed impaired Akt phosphorylation post-prandially or after insulin administration [124]. Compared to fish of the wild type, these fish exhibited noticeably higher fasting blood glucose levels as early as 3 months of age, a condition that was made worse by overfeeding. In another model, insulin resistance is achieved through liver specific knockdown of the insulin receptors using CRISPR-Cas9 [223].

Overfat accumulation in the liver can cause a variety of conditions, including cancer, fibrosis, and inflammation. These interrelated conditions are collectively known as NAFLD. Zebrafish fed an HFD, a high-cholesterol diet (HCD), a fructose diet (FD), overfeeding models, and starvation-induced models are among the dietary NAFLD models in this model [148]. Following a diet consisting of 13–16% soybean oil for 2–8 weeks, zebrafish exhibits a marked rise in plasma TG levels and lipid accumulation in their liver tissue [59, 190]. An additional, frequently utilised HFD containing 8% palmitic acid for 6 weeks, results in NAFLD, obesity, hyperlipidaemia, and a hyperglycaemic zebrafish phenotype [60]. On their side, following a HCD containing 5% cholesterol quickly induces a full NAFLD process, ranging from fatty liver to NASH in 2–3 weeks [122]. Short-term fructose consumption causes hepatic steatosis in larval zebrafish by displaying steatohepatitis symptoms such as mitochondrial abnormalities and endoplasmic reticulum defects [166]. Another popular model is overfeeding, which is accomplished by giving zebrafish more food or more often [89, 140]. Zebrafish are fed six times their typical diet intake in an overfeeding model, which mimics a high-calorie diet. The overfeeding diets have greater effects on the amino acid metabolism and autophagy activation of zebrafish [47, 230], and mRNA expressions of genes associated with lipogenesis and glucose metabolism, such as *lipin1*, *srebf1*, and *irs2a*, are decreased [47, 230]. Alternatively, zebrafish larvae that have been starved for 5–10 days post-fertilization after their yolk-supplied nutrition has been depleted undergo fasting treatment. This treatment promotes extrahepatic fatty acid uptake and *de novo* lipogenesis, which leads to hepatic steatosis [217]. Numerous mutant strains with liver phenotypes like NAFLD have been found using forward genetic screens or produced by reverse genetic techniques. A subunit of the transporter particle complex, which is involved in membrane traffic, exogenous biological metabolism, and endoplasmic reticulum stress, is encoded by the mutated gene *trappc11* in the *foigr* mutant. Due to the inhibition of lipid-linked oligosaccharide synthesis [164, 169], *foigr* mutant zebrafish exhibit a hepatomegaly phenotype and a reduction in protein N-glycosylation. This causes the unfolded protein response and the accumulation of secreted proteins in the endoplasmic reticulum, which in turn causes the formation of fatty liver [51, 56, 164]. The gene *cdipt* plays a vital role in the synthesis of phosphatidylinositol, the primary precursor of phosphoinositides, an essential modulator of protein secretion and calcium homeostasis. The pathological liver structure of *cdipt*-deficient zebrafish is comparable to that of NAFLD, with features such as enlarged liver, hepatocyte lipid accumulation and degeneration, elevated oxidative stress, and endoplasmic reticulum destruction [196]. Comprehensive research on the roles of genes, cell migration and differentiation, and the pathophysiology of illnesses like non NAFLD can be carried out using transgenic zebrafish, which can also be fluorescently labelled. Conditional expression of cannabinoid receptor 1 (*Cb1r*) in the liver of the transgenic line *Tg*(-*2.8fabp10a: Tet*^*off*^*-Cb1r-2 A-EGFP*)^*two23*^ promotes hepatic lipid accumulation and steatosis, which is important for food intake, weight gain, and various pathological features linked to obesity [142]. Activating transcription factor 4 (*Atf4*) regulates a number of metabolic illnesses. In the absence of doxycycline, the transgenic strain Tg(−−2.5actb1:Tetoff-Atf4-2 A-mCherry)zf2124 selectively expresses *Atf4*, which impacts intracellular and intravascular lipid accumulation as well as liver steatosis in zebrafish larvae by upregulating several genes involved in lipid biosynthesis and unfolded protein response activation. Moreover, giving transgenic fish an HFD can cause pathological alterations like NASH [220].

### *Drosophila melanogaster*

A wide range of human diseases can be studied using *Drosophila melanogaster* thanks to its structural and functional similarities to human organs, tissues, biological systems, and disease-related genes, quick generation times, and ability to perform large-scale genetic screens [14, 201].

Numerous strategies can result in DIO in drosophila larvae and adults. For high-carbohydrate feeding, high-sucrose diets are the most commonly used [27, 72, 86]; nonetheless, diets high in glucose and fructose have also been employed [162]. The most popular method for causing DIO in flies during HFD is supplementing with coconut oil [22, 87], though lard and the hydrogenated soybean and palm oil product known as Crisco have also been employed [109, 214]. In drosophila obesity studies, some researchers have recently started utilising an obesogenic high-sugar, HFD that is comparable to the one used in many rodent studies and is most likely the most similar to the standard Western obesogenic diet. Furthermore, obesity in flies can also be induced by genetic manipulations, which distinguish fat deposition from changes in blood sugar and the metabolism of carbohydrates [15]. Since flies carry approximately 75% of the genes known to be associated with human diseases, drosophila is useful for studying obesity because of its high genetic conservation with humans [157]. One of drosophila’s greatest advantages is its extensive genetic toolkit, which can be used to create genetic screens that find the genes required to regulate obesity. For instance, about 500 genes related to obesity were found in a genome-wide RNA interference screening. Among these were the drosophila orthologs of the glucagon, insulin, and TOR signalling pathways, which changed lean and obese phenotypes [150]. Amazingly, human orthologs exist for 62% of these obesity-related genes. Furthermore, new roles were found, such as hedgehog signalling’s bearing on the differentiation of white and brown adipocytes [133].

*Drosophila* diabetes models are currently being used extensively in studies of the mechanisms underlying the onset of diabetes and its complications. The high-carbohydrate diet has been shown to cause metabolic disorders in fruit fly larvae that are comparable to those seen in patients with T2DM, as Musselman et al. originally discovered in 2011 [143]. Additionally, other research demonstrates that adults fed high-carbohydrate diet al.so experience hyperglycaemia and insulin resistance [134]. In fruit flies, both adults and larvae, HFD feeding can result in elevated TG levels, which can cause problems with insulin and glucose metabolism. In addition, diabetes-related cardiac structural disease can result from both high-carbohydrate and HFD programmes. Research by Murillo-Maldonado et al. [136], Park et al. [144], and Tatar et al. [194] demonstrated that it is possible to cause insulin resistance in fruit flies by knocking out and knocking down key components of the insulin/TOR pathway. Mutants of *InR*,* chico*,* Dp110*,* Akt1*,* Rheb*, and *S6K*, for instance, disrupt the insulin/TOR pathway and, in turn, result in differing degrees of insulin resistance. These mutants show a series of T2DM phenotypes, such as developmental delay, small body size, significant lipid increase (obesity), and damage to brain and retina function.

Liver diseases are a burden in modern societies, particularly NAFLD and colon cancer. The central function of the liver in metabolic adaptation of flies is shared by the fat body and by the so-called oenocytes [201]. Whereas the fat body serves as an endocrine organ to coordinate metabolic homeostasis, it is also an important organ for glycogen and fat storage in flies [145]. Oenocytes, like hepatocytes in mammals, play a crucial role in the mobilisation and turnover of lipids. Lipid droplets are accumulated by oenocytes, indicating a close relationship between oenocytes and the fat body. This process is necessary for the mobilisation of lipids from the fat body. However, more research has revealed that oenocytes resemble mammalian hepatocytes in terms of their response to starvation than do fat body cells. Additionally, oenocytes express genes associated with hepatocyte differentiation, such as hepatocyte nuclear factor 4-a (*Hnf4-a*) and COUP transcription factor (*COUP-TF*), in addition to 22 homologs of human fat-metabolizing genes expressed in hepatocytes [82]. Lipid mobilization from the fat body during fasting produces lipid droplet accumulation in oenocytes, a metabolic change resembling hepatic steatosis, and the near distance between fat body and oenocyte helps to orchestrate this strong relationship between them. The fruit-fly model appears as a potent substitute for large-scale analyses because of the abundance of genetics tools available to control, in a tissue-specific manner, either over-expression or RNAi-inactivation of a gene of interest [201]. Stock centres offer collections of transgenic lines that target most drosophila genes, with an emphasis on orthologs of human genes related to disease [58]. Using this method, the roles of pertinent proteins were confirmed, including CDGSH-iron-sulfur-domain-containing-protein-2, whose haplo-insufficiency results in NAFLD and encourages the growth of colon cancer [176]. Moreover, this method revealed genes involved in fat deposition [150] and whose dysregulation in humans causes obesity, diabetes, and NAFLD [79]. To sum up, in vivo studies employing Drosophila in translational approaches will be helpful to validate enzymes and other molecules crucial for body homeostasis, increasing the chance to develop novel therapeutic strategies.

## Gastrointestinal simulators and colonic fermentation

In the field of nutrition, there is an in vitro tool that can be very helpful for the study of the compounds that appear along the digestive process: the gastrointestinal simulator. Contrary to the previous models, gastrointestinal simulators are not envisaged to study the effects of bioactive compounds on metabolic diseases. They are designed to simulate the digestive process and to obtain the metabolites that are generated in the stomach or the small intestine as a consequence of the process. The most usual method is INFOGEST [24], which can be completed in 7 days. It has an oral phase, gastric digestion that includes the use of gastric lipase, and intestinal digestion, which allows analyzing the release of micronutrients and the digestion products (e.g., simple sugars, amino acids/peptides, fatty acids).

Gastrointestinal simulators can vary from simple static models (like INFOGEST) to dynamic systems that incorporate gastrointestinal motility and other physiological factors. Some of these models incorporate also the colonic phase, in which microbiota from human donors can be added in order to study the modulation in gut composition that is exerted by different foods and digestive conditions, or the metabolites that are produced by the gut microbes after the fermentation process. They have potential applications for the study of prevention and/or treatment of diseases associated with intestinal dysbiosis. For example, the result of the different stages can be tested in other in vitro models such as cocultures of intestinal barrier or organoids. The following models are good examples:

- SIMGI (SIMulator Gastro-Intestinal) of Food Science Research Institute (Spain): An automated system that combines a gastric compartment with peristaltic mixing, a small intestinal reactor, and three-stage continuous reactors for simulating the colon [192].
- SHIME^®^ (Simulator of the Human Intestinal Microbial Ecosystem) of ProDigest BV, Belgium: A dynamic model that simulates the entire GI tract, including the stomach, small intestine, and both proximal and distal colon, with a focus on microbial communities [66].
- The Dynamic Gastrointestinal and Colonic Fermentation Model of AINIA (Spain): A model of five interconnected compartments that is computer-assisted for the automated control of the physiological conditions applied in colonic fermentation assays [160].

Table 1 summarizes the main features of the pre-clinical methodologies detailed in the review that are mostly used to investigate in the field of obesity and its associated metabolic diseases, particularly focusing on advantages and disadvantages of each model.

**Table 1:** Summary of the methodologies described in the review highlighting their positive aspects (advantages) and drawbacks (limitations)

Model	Tissue/organ	Advantages	Limitations	References
In vitro enzyme inhibition	Isolated enzymes (pancreatic lipase, α-amilase, α-glucosidase, dipeptidylpeptidase IV, protein tyrosine phosphatase 1B)	Precise control of experimental conditions and reproducibility Screening of compound libraries and large amount of samples Cost-effective and ethically accepted Mechanistic information	Bioavailability and bioaccesibility of substances is not considered Metabolites of tested substances are not evaluated Interference with absorbance or fluorescence Other non-enzymatic targets are involved in the bioactivity Commercial availability of recombinant, human or animal enzymes	[106, 111, 112, 120, 228]
Caco-2 cells	Intestinal barrier	Reproducibility Long time to achieve the differentiation process (21 days) Transport studies with the transwell^®^ system	Only 2D culture No representation of the cellular heterogeneity of the intestinal barrier Generation of colonic tight junction that do not represent tight junctions of the small intestine	[181]
Co-culture: Caco-2/HT29 MTX	Intestinal barrier	Generation of a mucus producing intestinal epithelium	Only 2D culture No representation of the cellular heterogeneity of the intestinal barrier	[20]
3D culture: Caco-2/HT29 MTX/Raji B	Intestinal barrier	Enhancement of intestinal metabolic activity Obtention of 3D villa-like structures	Complex experimental setup Difficulty to reproduce the intestinal motility	[8]
Intestinal cells connected with hepatic cells by microfluidic system	Intestinal barrier that integrates hepatic metabolism	Study of the first pass metabolism	Complex experimental setup Adsorption features of the tested drug or the generated metabolites by the biochip or the pipes of the system.	[23]
Humix^®^ system	Intestinal barrier that integrates microbiota	Integration of microbiota Access to some bacterial metabolites Enable human-microbial crosstalk	Complex experimental setup Difficulty to co-cultivate bacteria with intestinal cells	[171]
C2C12 cells, L6 cells	Skeletal muscle	Both differentiate into myotubes in user-controlled conditions. These models are reproducible and respond to metabolic stress atrophy. C2C12 are well-suited for studies on muscle development, regeneration, and differentiation L6 cells have high insulin-stimulated glucose uptake and oxidative capacities.	C2C12 and L6 cells are generally only used in 2D without their natural environment. Rodent cells which do not always perfectly reflect the human muscle physiology. C2C12 less sensitive to glucocorticoid-induced atrophy. L6 cells are less efficient at forming mature myotubes in serum-free media and less robust differentiation compared to C2C12.	[2, 46, 126]
Human primary culture of muscle progenitors	Skeletal muscle	The model can mimic donor’s phenotype.	Donor variability, low yield of muscle progenitors, finite proliferative capacity, possible dedifferentiation over time.	[12]
Immortalized human muscle stem cells	Skeletal muscle	Cells retain differentiation capacity and respond to therapeutical assays. Stable and could be implanted in diseased tissue.	Immortalization may lead to changes in gene expression and alter cell behavior and function: cells may acquire tumorigenic properties.	[127]
Myospheres (3D construct)	Skeletal muscle	Easy to create (self-assembled structure). Useful for high-throughput screening.	Poorly structured cell organization. Contain cells at different stages of maturation.	[53]
Myobundles (3D construct)	Skeletal muscle	Promotes the formation of aligned myotubes, closer to native muscle. Enables the study of muscle contraction force. Useful for co-cultures with motor neurons, endothelial cells…	Complex and costly manufacturing. Requires long maturation times to achieve functional muscle characteristics.	[4, 75]
Multiorgan-on-a-chip devices	Skeletal muscle and other tissues	Modeling of inter-organ interactions. Can integrate simulated blood flow, electrical stimulation and functional innervation.	High cost and complexity. Technology still emerging.	[69]
Human adipose derived mesenchymal stem cells (hADMSC)	Adipose tissue	Abundant, easy to isolate, inmunomodulatory, modifiable for therapeutic use	Donor and depot variability, potential tumorigenicity, unclear long-term safety, standardization challenges	[107, 121, 203]
Human adipose tissue explants	White adipose tissue (WAT)	Preserves 3D structure, ECM, and cellular heterogeneity (adipocytes, SVF, immune, endothelial cells) and maintains physiological interactions (paracrine, endocrine). Enables studies of inflammation, metabolism, angiogenesis etc. Suitable for human personalized research (age, sex, BMI, etc.).	Sample heterogeneity (subcutaneous/visceral) and collection methods affect reproducibility. High donor variability (age, sex, BMI, genetics). Viability/functionality decline over time in culture.	[35]
Spheroids	3D culture (cellular aggregates) Tumour-bacterial spheroids, full human spheroids (hepatocytes, cardiomyocytes, neuronal, adipose)	Functional versatility: replicate more accurately extracellular environment in vivo (cell-to-cell and cell-to-cell matrix interactions). Allow basic studies on cell-tissue function and can predict in vivo responses. Cultured alone or in combination and/or co-adjuvants. Small methodological variations allow the study of bacterial origin compounds of biological interest Broad inter-species development: murine & human cell types Extensive application: suitable for drug testing, prediction of treatment response (biomarkers), basic & translational studies	Can be developed with different methods which eventually lead to (small) physiological differences Careful selection of specific microenvironment conditions: aligned to the physiological milieu. Cell death (hypoxia & necrosis) in spheroid core Spheroid architecture pose a difficulty in equimolar distribution of bioactive compounds in global surface vs. spheroid core (osmolarity changes)	[21, 133, 154, 163]
Organoids	Liver, intestine, retina, lung	Physiologically more relevant than 2D models, able to reproduce human-specific biology advanced disease modeling capabilities by replicating pathological traits	Lack full organ complexity, technical challenges to reproduce microenvironment, high cost	[161, 218]
*C. elegans*	Whole organism	Multicellular model mimicking several human diseases. Simple structure and transparency, which allows to easily observe fat droplets and the digestive system. Cheap model, due to its small size, large number of progenies, and short life span. Existence of many low-cost mutant strains.	Very simply physiology, including the lack of a proper immune system, which limits the understanding of complex biological systems. There is not always a homologue for the entire mammalian genome. Its short life cycle implies that its results cannot always be extrapolated to long-lived organisms. *C. elegans* lacks adipocytes and is not able to synthesize cholesterol	[78, 174, 175, 213]
*Danio rerio* (Zebrafish)	Whole organism	Possess essential organs and regulatory mechanisms to control metabolism, like humans. Obesity models can be conveniently generated through overfeeding. Genetic models of obesity are available, including mutants and transgenic lines. Transgenic models can be fluorescently labelled to study gene function, cell migration, and pathophysiology.	Zebrafish leptin and leptin receptor differ from mammals, particularly in their expression patterns. Lack brown adipose tissue (BAT), which plays a crucial role in thermogenesis and energy expenditure in mammals. Difficult to track individual food intake: closely linked to growth rate and adipocyte formation.	[34, 61, 123, 165, 226]
*Drosophila melanogaster* (Fruit fly)	Whole organism	Many key metabolic pathways involved in lipid and carbohydrate metabolism are conserved between Drosophila and mammals. Drosophila has a robust genetic toolkit, allowing for easy manipulation of genes and pathways. Drosophila diets can be easily modified in the lab, making it a good model for studying dietary effects. Obesity can be induced through high-carbohydrate or high-fat diets.	Evolutionarily distant from humans. Has a fat body that combines functions of the liver and adipose tissue of mammals: lacks a pancreas as a distinct organ; neurosecretory cells in the brain produce insulin-like peptides. Cholesterol auxotroph, meaning it cannot synthesize cholesterol de novo, unlike mammals.	[14, 25, 102, 157, 201]
Gastrointestinal simulators	Gastrointestinal tract	Helpful for the study of the compounds that appear along the digestive process or the colonic fermentation of foods. The colonic phase allows to analyze the shifts in microbiota composition and function.	It is difficult to perfectly simulate the physiological conditions of the human gastrointestinal tract. Gut microbes must accommodate to the in vitroconditions, and it implies the death/decay of many species.	[24, 66, 160, 192]

## Conclusion

The present review summarizes current knowledge and future advances on the most relevant methodologies and experimental models to perform scientific research on metabolic disturbances, particularly, obesity and diabetes, and the study of the effects and mechanisms of bioactive compounds. We provide constructive overview of the main characteristics of these methodologies, ranging from basic in vitro models (enzyme assays, 2D, 3D cell cultures) and pluricellular (*Caenorhabditis elegans*, *Drosophila melanogster*, *Danio rerio*) models, highlighting their advantages and disadvantages, usefulness and limitations as well as current and future applications. Taking advantage of the multiple and variable in vitro models (enzyme assays, standard 2D cell cultures and co-cultures, 3D cultures), all these allow a more translational and ethically close approach that minimizes animal testing, although in vivo pre-clinical models will still be needed. Despite some caveats, developing novel and complementary methodologies to those presented herein would be of main interest in health science in coming years. Using and applying diverse and advanced experimental settings would boost scientific outcomes in biomedical and nutrition fields paving the way to precision medicine and personalized nutrition.

## Data Availability

No datasets were generated or analyzed during the current study.

## Declarations

### Clinical trial number

N/A.
